# 3% diquafosol sodium combined with 0.1% fluorometholone for postoperative dry eye in pterygium patients with preoperative tear deficiency: a pilot retrospective study

**DOI:** 10.3389/fmed.2026.1859002

**Published:** 2026-06-30

**Authors:** Qin Tian, Xingde Liu, Zhangquan Peng, Jiaqian Li, Juan Xie, Dan Zhang, Juan Du

**Affiliations:** 1 Department of Ophthalmology, Ziyang Central Hospital (West China Hospital of Sichuan University Ziyang Hospital), Ziyang, Sichuan, China; 2 The First Affiliated Hospital of Chongqing Medical University, Chongqing, China

**Keywords:** diquafosol sodium, fluorometholone, pterygium, postoperative dry eye, tear insufficiency, retrospective study

## Abstract

**Background:**

To evaluate the clinical efficacy and safety of 3% diquafosol sodium combined with 0.1% fluorometholone for postoperative dry eye in pterygium patients with preoperative tear deficiency.

**Methods:**

This single-center retrospective study included 82 pterygium patients (82 eyes) with preoperative tear deficiency who underwent pterygium excision combined with limbal stem cell transplantation. Patients were divided into an observation group (diquafosol + fluorometholone, *n* = 42) and a control group (sodium hyaluronate + fluorometholone, *n* = 40). Tear break-up time (BUT), corneal fluorescein staining (CFS) score, Ocular Surface Disease Index (OSDI), Schirmer *I* test (SIt), and intraocular pressure (IOP) were assessed preoperatively and at 2 and 4 weeks postoperatively. Adverse events and complications were recorded.

**Results:**

Eighty patients completed follow-up (41 in observation group, 39 in control group). At 4 weeks postoperatively, the observation group showed significantly longer BUT, lower CFS and OSDI scores, and higher SIt values compared to the control group (all *p* < 0.05). No significant intergroup differences in IOP were observed at any time point. Transient ocular stinging occurred in 2 patients (4.88%) in the observation group, with no severe complications in either group.

**Conclusion:**

In this pilot study, the combination of 3% diquafosol sodium and 0.1% fluorometholone showed preliminary evidence of improving tear film stability, repairing ocular surface damage, and alleviating dry eye symptoms in pterygium patients with preoperative tear deficiency, without significantly affecting IOP. Its efficacy appeared superior to sodium hyaluronate combined with fluorometholone. These findings support further investigation in larger, longer-term randomized controlled trials.

## Introduction

Pterygium is a prevalent fibrovascular proliferative disorder of the ocular surface (1–3). It is pathologically characterized by the invasion of fibrovascular tissue from the bulbar conjunctiva toward the central cornea (4), which compromises corneal optical integrity and disrupts homeostasis of the ocular surface microenvironment. Pterygium excision combined with autologous limbal stem cell transplantation represents a standard surgical procedure. However, surgical trauma inevitably exacerbates ocular surface inflammation, destabilizes the tear film, and induces or aggravates postoperative dry eye symptoms (5). Patients with preoperative tear deficiency carry a significantly higher risk of moderate-to-severe postoperative dry eye (6). The global prevalence of dry eye disease has been progressively increasing, with its reported incidence following pterygium surgery ranging from 30 to 50%. Patients with preoperative abnormal tear secretion often present with more severe and prolonged symptoms (7–9). The core pathophysiological mechanism involves insufficient tear production secondary to lacrimal gland hypofunction. Surgical transection of conjunctival nerves further impairs tear secretion (10). In the absence of timely intervention, ocular surface damage may deteriorate and even progress to refractory dry eye (11). Conventional treatment relies on artificial tears combined with glucocorticoids. Sodium hyaluronate merely provides exogenous tear supplementation, whereas fluorometholone exerts anti-inflammatory effects (12); however, neither agent promotes endogenous tear secretion, resulting in limited efficacy in patients with preoperative lacrimal gland hypofunction (13, 14). As a selective P2Y₂ receptor agonist, diquafosol sodium stimulates secretion of both the aqueous and mucin components of tears and has been reported to exhibit superior efficacy compared with artificial tears alone (15, 16). Theoretically, combined administration of diquafosol sodium and fluorometholone may produce synergistic effects. However, the clinical value of this combination in pterygium patients with preoperative tear dysfunction remains unclear. Accordingly, the present study was designed to evaluate the efficacy and safety of diquafosol sodium plus fluorometholone in alleviating postoperative dry eye in this high-risk population, thereby providing a reference for the optimization of perioperative ocular surface protection.

## Methods

### Ethics approval and consent to participate

This study was approved by the Ethics Committee of Ziyang Central Hospital, West China Hospital of Sichuan University (2024. No. 022). All participants provided written informed consent before enrollment.

### Participants

#### Inclusion criteria

(1) Diagnosed with primary pterygium and scheduled for or underwent pterygium excision combined with autologous limbal stem cell transplantation.
(2) Preoperative Schirmer *I* test (SIt) ≤ 10 mm/5 min, confirming tear secretion insufficiency.
(3) Occurrence of dry eye symptoms (dryness, foreign body sensation, burning sensation, blurred vision) within 1 week postoperatively, accompanied by BUT <10 s and CFS score ≥1 point.
(4) Able to comply with regular follow-up for at least 4 weeks after surgery.

#### Exclusion criteria

(1) Complicated with other ocular surface or intraocular disorders.
(2) History of previous ocular surgery or penetrating ocular trauma.
(3) Long-term use of medications that may affect tear secretion prior to surgery.
(4) Hypersensitivity or allergy to any of the study medications.
(5) Development of severe postoperative complications that rendered outcome evaluation unfeasible.
(6) Loss to follow-up or incomplete clinical data.

### Study design and bias control

This was a single-center retrospective chart review study. Patients were allocated to two groups based on the actual postoperative topical medication regimens they received, rather than through a process of randomization. To minimize bias, the following measures were implemented: (1) all clinical data were extracted from electronic medical records using coded patient identifiers without treatment group information; (2) statistical analysis was performed by an independent statistician who was not involved in patient management or outcome assessment; (3) all outcome measurements followed standardized protocols as described below.

### Procedures

All patients underwent pterygium excision combined with limbal stem cell transplantation. All procedures were performed by the same experienced ophthalmic surgeon in accordance with a standardized surgical protocol. After complete excision of pterygium tissue, a conjunctival graft containing limbal stem cells was harvested from the superior or superotemporal quadrant of the ipsilateral eye and secured to the exposed scleral bed. Routine anti-infective therapy was administered postoperatively.

This was a retrospective chart review study. Patients were allocated to two groups based on the actual postoperative topical medication regimens they received, rather than through a process of randomization. The observation group received 3% diquafosol sodium eye drops combined with 0. 1% fluorometholone eye drops. The control group received 0. 1% sodium hyaluronate eye drops combined with 0. 1% fluorometholone eye drops. Both groups received 0.1% fluorometholone eye drops with the following tapering schedule: four times daily during the first postoperative week, three times daily during the second week, twice daily during the third week, and once daily during the fourth week. The dosage frequency, treatment duration, and tapering schedule were identical between the two groups.

All patients underwent standardized follow-up examinations at 2 and 4 weeks postoperatively. Tear film break-up time (BUT), corneal fluorescein staining (CFS) score, Ocular Surface Disease Index (OSDI) score, Schirmer *I* test (SIt) value, and intraocular pressure (IOP) were evaluated. Adverse events including ocular irritation, conjunctival hyperemia, and IOP elevation, as well as other postoperative complications, were monitored and documented.

### Outcomes

1 Ocular surface function parameters

Tear film break-up time (BUT), corneal fluorescein staining (CFS) score, Schirmer *I* test (SIt), and Ocular Surface Disease Index (OSDI) score were measured preoperatively and at 2 and 4 weeks postoperatively to evaluate the improvement of dry eye.

#### Detailed procedures for tear function tests

Tear break-up time (BUT): After instillation of 5 μL of 1% sodium fluorescein into the inferior conjunctival fornix, the patient was instructed to blink naturally several times to distribute the dye evenly. The time from the last complete blink to the first appearance of a dry spot or breakup line on the cornea was measured using a slit-lamp with a cobalt blue filter. The measurement was repeated three times, and the average value was recorded in seconds.

Schirmer *I* test: The test was performed without topical anesthesia. A standard sterile Schirmer strip (Whatman No. 41 filter paper) was folded at the notch and placed at the junction of the middle and lateral one-third of the lower eyelid margin. The patient was asked to close the eyes gently for 5 min. After removal, the length of wetting from the notch was measured in millimeters.

Justification: While non-invasive methods for tear film assessment are increasingly available, the BUT and Schirmer *I* test remain the most widely used and validated objective measures for dry eye disease in routine clinical practice. They are recommended by the TFOS DEWS II diagnostic methodology report and are considered standard in settings without access to advanced imaging equipment. Our hospital currently does not have non-invasive tear film imaging systems, and these tests were performed strictly according to standardized protocols to ensure reliability.

2 Intraocular pressure (IOP)

monitoringIntraocular pressure was measured at the same time points to assess IOP changes and rule out steroid-induced ocular hypertension during treatment.

3 Safety indicators

Adverse ocular reactions were recorded, including ocular stinging, foreign body sensation, conjunctival hyperemia, itching, and blurred vision. Severe postoperative complications such as infection, graft detachment, poor wound healing, and pterygium recurrence were also documented, and the incidence was calculated.

4 Assessment consistency and blinding

All pre- and postoperative clinical parameters (BUT, CFS, SIt, OSDI, and IOP) were measured by the same two trained ophthalmologists following a standardized protocol throughout the study period. Due to the retrospective design, the outcome assessors were not blinded to the treatment allocation.

### Statistical analysis

Statistical analysis was performed using IBM SPSS Statistics 26.0 software. Measurement data were tested for normality using the Shapiro–Wilk test. Normally distributed data were presented as mean ± standard deviation (±s). Between-group comparisons were conducted using independent-sample *t*-tests, and within-group comparisons between preoperative and postoperative time points were performed using paired *t*-tests. Non-normally distributed data were expressed as median (interquartile range) and analyzed using the Mann–Whitney *U* test. Count data were reported as cases (percentage) [*n*(%)] and compared using the *χ*^2^ test or Fisher exact test, as appropriate. A *p*-value < 0.05 was considered statistically significant.

## Results

**Figure 1:**
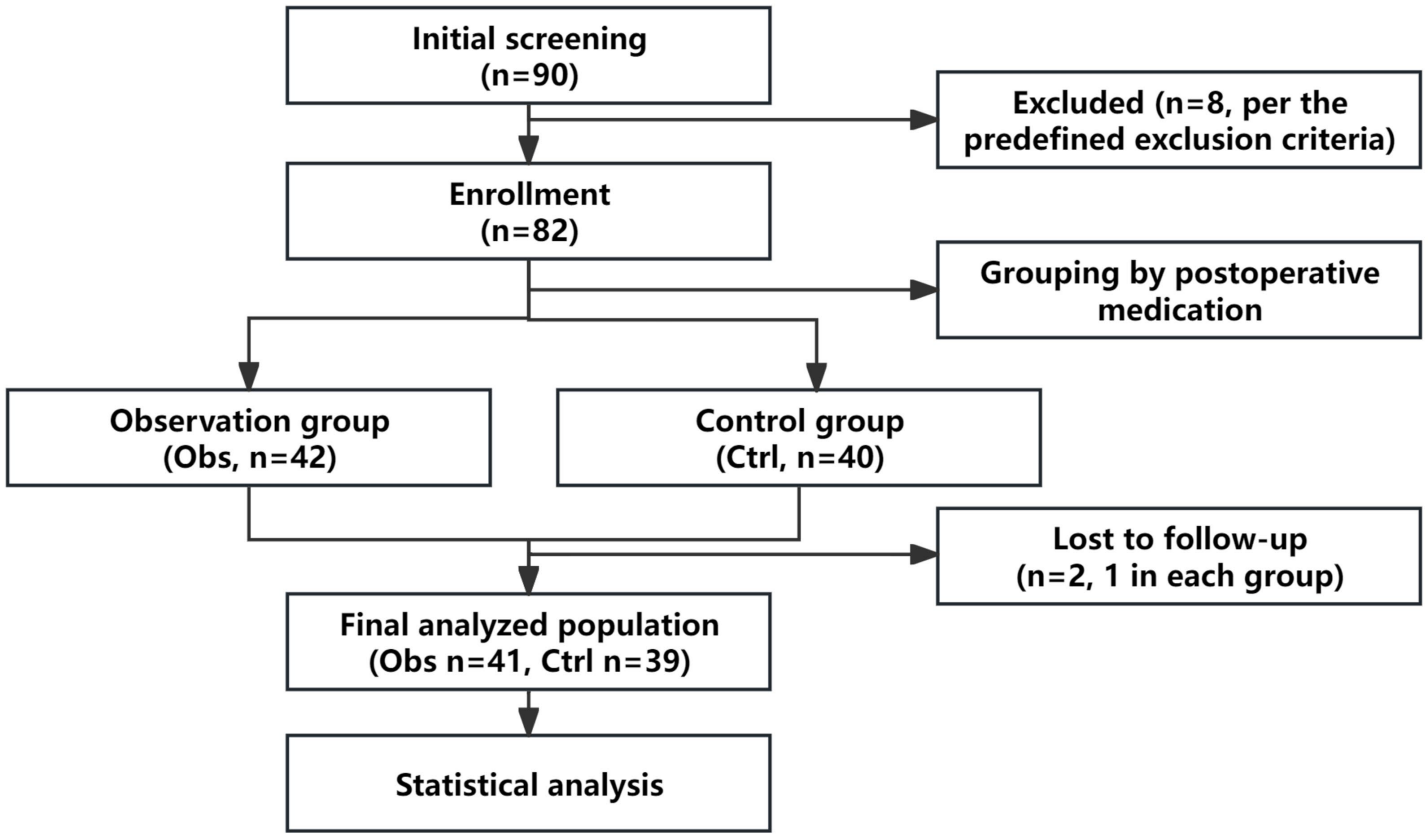
Study flow chart of retrospective cohort study for case enrollment.

A total of 82 patients (82 eyes) with primary pterygium and preoperative tear deficiency were enrolled. All patients underwent pterygium excision combined with limbal stem cell transplantation. According to postoperative medication regimens, patients were divided into the observation group (diquafosol sodium combined with fluorometholone) and the control group (sodium hyaluronate combined with fluorometholone). One patient was lost to follow-up in each group, leaving 41 eyes in the observation group and 39 eyes in the control group for final analysis. The overall follow-up completion rate was 97.56%. The study flowchart is presented in Figure 1. Baseline characteristics of the two groups are summarized and compared in Table 1. No statistically significant differences were observed in baseline demographic and clinical characteristics between the two groups (all *p* > 0.05), including sex, age, pterygium size, and disease duration. Similarly, there were no significant intergroup differences in preoperative ocular surface parameters and intraocular pressure (all p > 0.05), including BUT, CFS score, SIt value, OSDI score, and intraocular pressure.

**Table 1:** Baseline demographics and clinical data of the two groups.

Parameter	Observation group (*n* = 41)	Control group (*n* = 39)	*p*-value
Gender (male/female)	18/23	19/20	0.823^†^
Age (years)	56.18 ± 7.92	55.87 ± 8. 12	0.863^*^
Pterygium size (mm)	3.20 ± 0.87	3. 18 ± 0.90	0.920^*^
Pterygium duration (years)	2.33 ± 0.95	2.28 ± 0.87	0.807^‡^
Preoperative BUT (s)	10.32 ± 1.45	10.28 ± 1.39	0.900^*^
Preoperative CFS score (points)	0.84 ± 0.43	0.82 ± 0.45	0.839^*^
Preoperative SIt (mm/5 min)	7.23 ± 1.65	7.18 ± 1.59	0.891^*^
Preoperative OSDI score (points)	8.70 ± 2.18	8.65 ± 2.08	0.917^*^
Preoperative IOP (mmHg)	15.30 ± 2.18	15.28 ± 2.20	0.968^*^

^*^Independent-sample *t*-test; ^†^based on Chi-square test/Fisher’s exact test; ^‡^based on Mann–Whitney *U* test.

### Changes in ocular surface function and intraocular pressure

As shown in Table 2, at 2 weeks postoperatively, the observation group exhibited significantly longer BUT, lower CFS and OSDI scores, and higher SIt values compared with the control group (all *p* < 0.05). At 4 weeks postoperatively, the observation group still showed significantly longer BUT, lower CFS score, and higher SIt value than the control group (all *p* < 0.05).

**Table 2:** Comparison of ocular surface function and intraocular pressure between the two groups.

Parameter	Observation group (*n* = 41)	Control group (*n* = 39)	*p*-value
BUT(s)			
Preop	10.32 ± 1.45	10.28 ± 1.39	0.900^*^
Postop (2w)	8.93 ± 1.58	4.83 ± 1.15	*p* < 0.001^*^
Postop (4w) CFS (points)	10.26 ± 1.34	7. 18 ± 1.26	*p* < 0.001^*^
Preop	0.84 ± 0.43	0.82 ± 0.45	0.839^*^
Postop (2w)	1.01 ± 0.32	2.62 ± 0.52	*p* < 0.001^*^
Postop (4w)	0.82 ± 0.34	1.57 ± 0.48	*p* < 0.001^*^
SIt (mm/5 min)			
Preop	7.23 ± 1.65	7. 18 ± 1.59	0.891^*^
Postop (2w)	10.51 ± 2.16	7.84 ± 1.78	*p* < 0.001^*^
Postop (4w) OSDI (points)	11.89 ± 2.05	8.02 ± 1.83	*p* < 0.001^*^
Preop	8.70 ± 2.18	8.65 ± 2.08	0.917^*^
Postop (2w)	25.61 ± 6.42	40.68 ± 10.32	*p* < 0.001^*^
Postop (4w) IOP (mmHg)	10.57 ± 3.24	24. 19 ± 3.87	*p* < 0.001^*^
Preop	15.30 ± 2.18	15.28 ± 2.20	0.968^*^
Postop (2w)	15.18 ± 2.30	15.12 ± 2.13	0.904^*^
Postop (4w)	14.39 ± 2.11	14.32 ± 2.07	0.881^*^

^*^Independent-sample *t*-test.

Intraocular pressure in both groups remained within normal limits at 2 and 4 weeks after surgery, with no significant intergroup differences at any time point (*p* > 0.05). No cases of steroid-induced ocular hypertension were noted in either group.

### Adverse reactions and postoperative complications in the two groups

As shown in Table 3, during the treatment period, transient ocular stinging occurred in 2 eyes out of 41 (4.88%) in the observation group, with no other discomfort. These symptoms resolved spontaneously 3–5 days after medication administration without special intervention. None of the 39 eyes in the control group developed obvious adverse reactions, including ocular stinging, conjunctival congestion, aggravated dryness, or elevated intraocular pressure.

**Table 3:** Adverse reactions and postoperative complications in the two groups.

Adverse reactions/complications	Observation group (*n* = 41)	Control group (*n* = 39)	*p*-value
Adverse reactions			
Transient ocular stinging	2 (4.88%)	0 (0.00%)	*p* > 0.05^†^
Conjunctival congestion	0 (0.00%)	0 (0.00%)	–
Aggravated dryness	0 (0.00%)	0 (0.00%)	–
Elevated IOP	0 (0.00%)	0 (0.00%)	–
Severe complications			
Surgical site infection	0 (0.00%)	0 (0.00%)	–
Active bleeding	0 (0.00%)	0 (0.00%)	–
Corneal perforation	0 (0.00%)	0 (0.00%)	–
Early pterygium recurrence	0 (0.00%)	0 (0.00%)	–
Wound healing	41 (100.00%)	39 (100.00%)	–

^†^Based on Chi-square test/Fisher’s exact test.

During postoperative follow-up, all patients achieved smooth recovery with bilateral topical levofloxacin eye drops for prophylactic infection control. No severe complications occurred in either group, including surgical site infection, active bleeding, corneal perforation, or early pterygium recurrence. All surgical wounds healed uneventfully.

## Discussion

Pterygium excision combined with limbal stem cell transplantation is the standard surgical approach for pterygium. However, postoperative dry eye is especially severe in patients with preoperative tear deficiency. Owing to inadequate basal tear secretion and postoperative ocular surface injury, these patients are prone to moderate-to-severe dry eye, which not only exacerbates ocular discomfort and causes visual fluctuations but also delays corneal epithelial healing and increases the risk of pterygium recurrence. Conventional treatments currently mainly involve topical artificial tear replacement and short-term anti-inflammatory therapy, which rarely correct endogenous tear deficiency fundamentally, resulting in unsatisfactory recovery of ocular surface function in some high-risk patients. Therefore, exploring optimized therapeutic strategies that can simultaneously improve abnormal tear dynamics and control inflammatory responses is of great clinical importance.

This study was a single-center retrospective analysis enrolling pterygium patients with preoperative tear deficiency, aiming to systematically evaluate the clinical efficacy of 3% diquafosol sodium combined with 0. 1% fluorometholone versus conventional sodium hyaluronate therapy. This combined regimen exerts synergistic effects via the tear secretagogue action of diquafosol sodium and the anti-inflammatory effect of fluorometholone. Our results demonstrated that this strategy significantly improved tear film stability, repaired ocular surface damage, and increased basal tear secretion, without an increased risk of elevated intraocular pressure. We herein analyzed the role of its dual “secretagogue plus anti-inflammatory” mechanism in short-term postoperative outcomes and discussed its application prospects in the standardized management of postoperative dry eye.

The observation group exhibited better tear film stability and ocular surface repair in the early postoperative period, which was mainly attributed to the specific activation of P2Y₂ receptors by diquafosol sodium. Diquafosol sodium promotes the secretion of chloride ions and water from conjunctival epithelial cells and induces mucin release from goblet cells, thereby reconstructing the three-layer structure of the tear film at the source. This represents a fundamental difference from sodium hyaluronate, which only provides exogenous supplementation. Previous studies have confirmed that diquafosol sodium has unique advantages in promoting endogenous tear secretion and shows superior efficacy in moderate-to-severe dry eye accompanied by inflammation (17).

In the present study, ocular surface inflammation induced by surgical trauma further suppressed residual tear secretion, which was effectively counteracted by the secretagogue effect of diquafosol sodium, resulting in better efficacy than high-molecular-weight sodium hyaluronate that only offers lubrication (18). Research on dry eye after cataract surgery also demonstrated that diquafosol sodium is superior to sodium hyaluronate in improving tear secretion as measured by the Schirmer test, which is consistent with our findings at 4 weeks postoperatively (19).

In conclusion, this combined regimen can restore the dynamic balance of the tear film more efficiently in the early postoperative period via the above dual mechanism. In terms of subjective symptom relief and ocular surface injury repair, the combined therapy effectively broke the vicious cycle of “inflammation–dryness.” Fluorometholone inhibited early postoperative non-specific inflammation, reduced stimulation of corneal nerve endings by inflammatory mediators, and lowered OSDI scores; meanwhile, diquafosol sodium thickened the mucin layer, enhanced tear film adhesion, and accelerated corneal epithelial repair. Studies have demonstrated that sodium hyaluronate is less effective than diquafosol sodium in upregulating Muc5AC mRNA expression, which may explain the more rapid improvement of corneal fluorescein staining scores observed in the observation group. Consistently, a large-scale population study indicated that traditional artificial tears fail to address the fundamental issue of insufficient secretion, leading to recurrent symptoms (20).

In this study, OSDI scores in the observation group decreased significantly at 2 weeks postoperatively, suggesting that mechanism-based therapy provides more rapid symptom relief than simple replacement therapy, especially during the sensitive period of postoperative visual fluctuation. This regimen achieves synchronous improvement in subjective symptoms and objective signs by synergistically regulating the ocular surface microenvironment and promoting epithelial healing.

Regarding safety, diquafosol sodium combined with tapering-dose fluorometholone did not increase the risk of abnormal intraocular pressure, and most adverse events were transient and self-limiting, providing a reliable safety profile for clinical application. Transient ocular stinging related to diquafosol sodium resolved spontaneously without compromising medication compliance. The weekly tapering regimen of fluorometholone effectively prevented steroid-induced ocular hypertension, which was consistent with safety findings in previous literature. Importantly, because both groups received the identical fluorometholone tapering regimen, any potential effect of the corticosteroid on tear film properties or lacrimal gland function was balanced between groups, allowing a fair comparison of the added effect of diquafosol sodium versus sodium hyaluronate. Although sodium hyaluronate exhibited favorable safety, its efficacy was limited in patients with marked tear deficiency. No severe complications occurred in the observation group, and there were no significant intergroup differences in intraocular pressure. These results confirm that the combined regimen is safe and feasible for the refined management of postoperative dry eye.

This study has several limitations. The sample size was relatively small and the follow-up period was short (only up to 4 weeks postoperatively), which reduced statistical power and limited the assessment of secondary outcomes and rare adverse events. Furthermore, the lack of blinding of outcome assessors, inherent to the retrospective design, may introduce potential detection bias, although standardized measurement protocols were strictly followed. Long-term outcomes, such as dry eye recurrence, lacrimal gland function, and ocular surface homeostasis after drug cessation, could not be evaluated. The use of traditional BUT and Schirmer tests, rather than non-invasive methods, is another limitation; however, these tests remain clinically validated and were performed under strict protocols. Given the short follow-up, this study should be considered a pilot/preliminary study. The data provide a basis for sample size calculation and design of future large-scale, long-term prospective randomized controlled trials. Moreover, combined detection of tear biomarkers may further clarify the molecular mechanisms underlying the promotion of endogenous tear secretion.

## Data Availability

The original contributions presented in the study are included in the article/supplementary material, further inquiries can be directed to the corresponding authors.
